# Supplementary material for: Computational Modeling of the Crosstalk Between Macrophage Polarization and Tumor Cell Plasticity in the Tumor Microenvironment
Source: Front Oncol. 2019 Jan 23;9:10. doi: 10.3389/fonc.2019.00010 (PMC6351454; doi:10.3389/fonc.2019.00010)
Supplement: Supplementary file 1 [file Data_Sheet_1.pdf]

Supplementary materials: Computational modeling of the crosstalk between macrophage polarization and tumor cell plasticity in the tumor microenvironment

Xuefei Li[1†], Mohit Kumar Jolly[1†‡], Jason T. George[1,2,3], Kenneth J. Pienta[4], Herbert Levine[1,2,5,6,#]

[1]Center for Theoretical Biological Physics, Rice University, Houston, Texas, United States
[2]Department of Bioengineering, Rice University, Houston, Texas, United States
[3]Medical Scientist Training Program, Baylor College of Medicine, Houston, Texas, United States
[4]The James Buchanan Brady Urological Institute, Johns Hopkins University School of Medicine, Baltimore, Maryland, United States
[5]Department of Physics and Astronomy, Rice University, Houston, Texas, United States
[6]Department of Physics, Northeastern University, Boston, Massachusetts, United States

[†]These authors equally contributed to this work
[‡]Current address: Centre for BioSystems Science and Engineering, Indian Institute of Science, Bangalore, India

[#]To whom correspondence should be sent:
Herbert Levine: herbert.levine@rice.edu

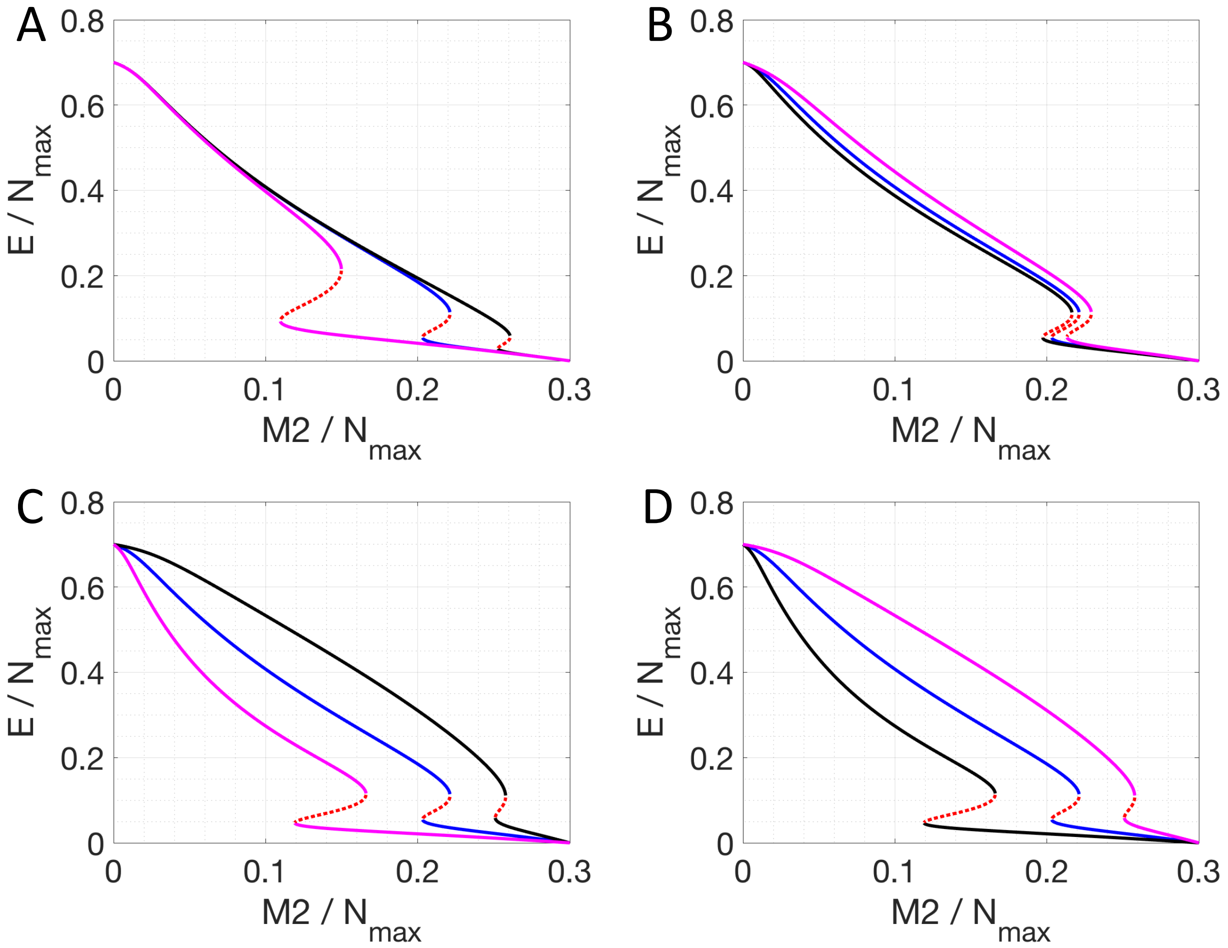

Figure S1 **Parameter-sensitivity analysis for K^0_E (A), K^0_M (B), η_{em} (C), η_{me} (D)**. Blue solid lines are the steady-state solutions with default parameters: K^0_E=0.1, K^0_M=0.1, η_{em}=1/72 h^{-1}, and η_{me} =1/120 h^{-1}. Black and cyan solid lines are steady-state solutions with half and twice of the corresponding parameters.

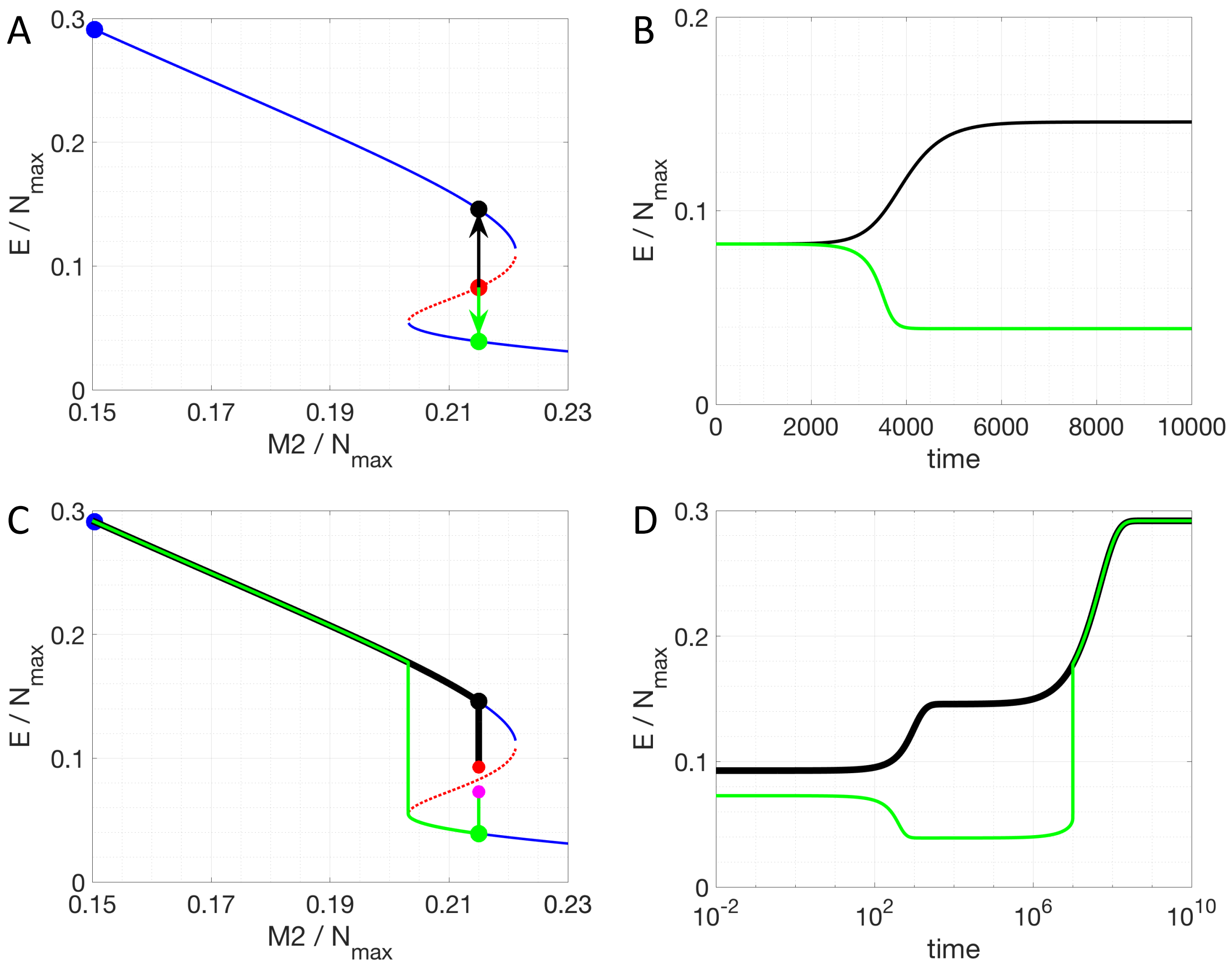

Figure S2 **Evolution of epithelial and M_2 populations in Model I and effects of the perturbation on M_1-M_2 interconversion.** A. Steady-state solutions of epithelial and M_2 populations of Model I are shown in blue solid (stable) and red dotted (unstable) lines. To illustrate the steady states on the red dotted line are unstable, we perturb the epithelial population from one steady state (the red dot) by a small number ($+/-10^{-6}$). Depending on the value of perturbation, the system will evolve to state I (black dot) or state II (green dot), respectively. The corresponding time evolution of the epithelial population is shown in B. The black and green lines are the evolution for the perturbation with 10^{-6} and -10^{-6}, respectively. C. Effects of the perturbation on M_1-M_2 interconversion. If we perturb both η_{12} and η_{21} from 0 to10^{-6}, the evolution trajectories of epithelial and M_2 populations are shown as black and green lines. The only one steady state of the system after this perturbation is represented by the blue dot. If the initial condition is assigned as the red and magenta dot, the E and M_2 population will evolve following the black and green lines, respectively. The corresponding time evolution of the epithelial population are shown in D.

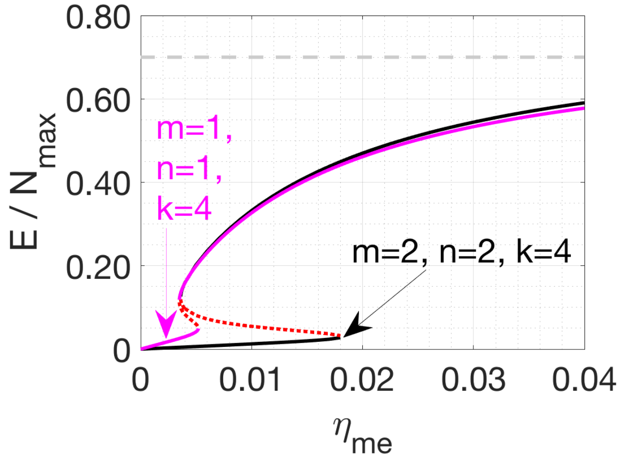

Figure S3 **Narrowed bi-stable region with smaller m and n for Model II.** Black solid lines are the steady-state solutions for Model II with m=2, n=2, and k=4, whereas cyan color lines represent steady-state solutions for Model II with m=1, n=1, and k=4. The bi-stable region (red dotted lines) is narrow for the case with m=1, n=1, and k=4.

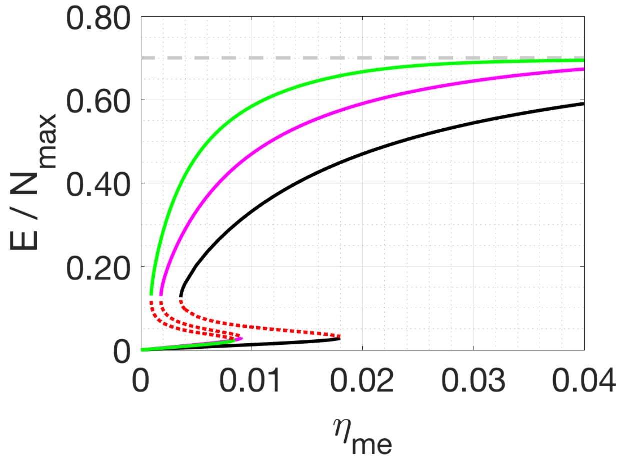

Figure S4 **Lowered threshold of η_{me} for Model II by varying parameters.** Black solid lines are the steady-state solutions with η_{12}=η_{21}=1/72 h^{-1} and η_{em}=1/72 h^{-1}; cyan color lines represent steady-state solutions with η_{12}=η_{21}=1/72 h^{-1} and η_{em}=1/144 h^{-1}; green lines are steady-state solutions with η_{12}=1/144 h^{-1}, η_{21}=1/72 h^{-1} and η_{em}=1/72 h^{-1}or η_{12}=1/72 h^{-1}, η_{21}=1/36 h^{-1} and η_{em}=1/72 h^{-1}.

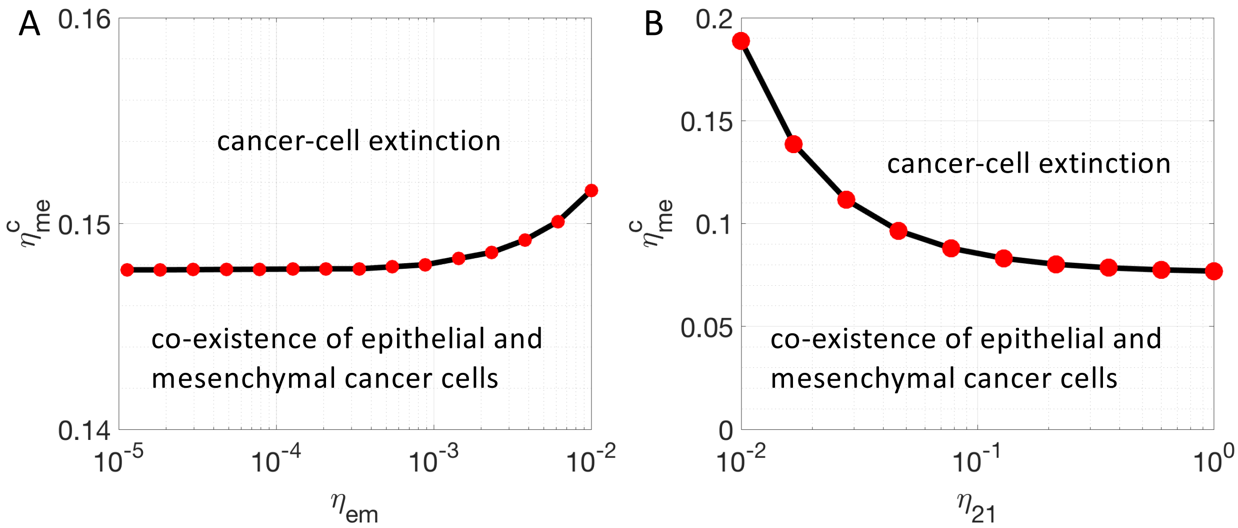

Figure S5 **Critical value η^{c}_{me} as a function of η_{em} or η_{21}.** For a given η_{em} (A) or η_{21} (B), once η_{me} goes across η^{c}_{me}, the cancer cell extinction state becomes the only stable steady state of the system. The critical value η^{c}_{em} is plotted as a function of η_{em} (A) or η_{21} (B). The default parameters are as shown in Table 2. In A, lower η_{em} gives rise to lower η^{c}_{me}, but there is a lower limit of η^{c}_{me} even for very small η_{em}. In B, higher η_{21} gives rise to lower η^{c}_{me}, but there is a lower limit of η^{c}_{me} even for very high η_{21}. In the current parameter setting, η^{c}_{me} will not be reduced the estimated experimental value (~0.01 h^{-1}) by varying η_{em} or η_{21}.

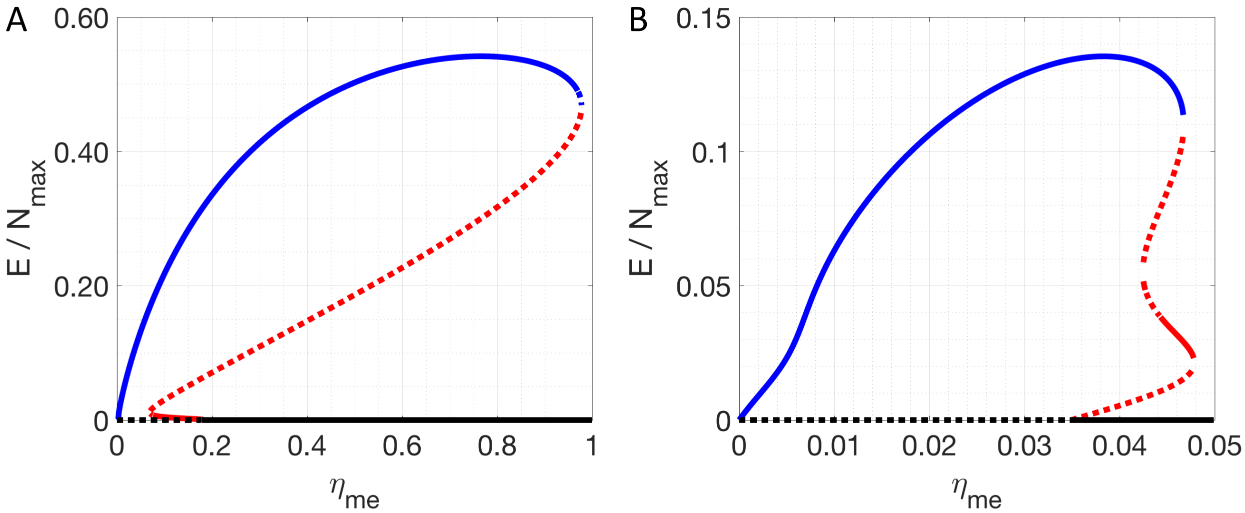

Figure S6 **Effects of changing M_c in Model III.** A. M_c=0.1. B. M_c=0.5. The default M_c used in Model III equals to 0.3. Generally, a higher M_c shifts the transition point, after which the only stable steady state of the system is the cancer-extinction state, to a lower value, though the stability of steady states in each branch might differ from the case where M_c=0.3.

	Adeno COL		Adeno PCA		BCA Invasive		Lung Adeno	
M2 Gene	p-value	95% CI	p-value	95% CI	p-value	95% CI	p-value	95% CI
'AOAH'	2.27E-05	[0.41 1.09]	3.92E-06	[0.27 0.66]	1.25E-15	[0.49 0.80]	3.30E-16	[0.76 1.23]
'ARHGAP24'	1.57E-03	[0.15 0.63]	3.05E-19	[0.49 0.76]	4.94E-36	[0.54 0.74]	1.29E-08	[0.29 0.58]
'CCRL2'	3.06E-04	[-0.45 -0.13]	8.25E-05	[0.12 0.36]	2.88E-07	[0.17 0.37]	4.35E-06	[0.26 0.65]
'CD38'	1.83E-02	[0.07 0.77]	4.81E-02	[0.00 0.85]	1.71E-05	[0.25 0.67]	2.86E-02	[0.03 0.57]
'CFB'	4.81E-04	[-0.69 -0.20]	3.10E-02	[0.03 0.67]	ns	[-0.18 0.34]	ns	[-0.28 0.26]
'CP'	2.96E-03	[0.23 1.11]	ns	[-0.13 0.62]	2.55E-02	[0.05 0.81]	ns	[-0.16 0.59]
'CPD'	ns	[-0.17 0.07]	ns	[-0.10 0.12]	ns	[-0.08 0.14]	2.09E-05	[0.21 0.57]
'CXCL10'	1.25E-02	[0.12 1.00]	ns	[-0.32 0.19]	1.86E-04	[0.21 0.66]	1.06E-12	[0.82 1.42]
'DDX58'	4.71E-04	[0.15 0.51]	ns	[-0.06 0.19]	ns	[-0.06 0.17]	1.80E-05	[0.17 0.44]
'DDX60'	ns	[-0.17 0.33]	6.03E-03	[0.07 0.41]	2.85E-02	[0.02 0.31]	4.25E-10	[0.41 0.78]
'EBI3'	1.70E-02	[0.05 0.54]	2.93E-03	[0.08 0.39]	7.46E-10	[0.30 0.57]	9.97E-11	[0.52 0.95]
'F11R'	6.89E-05	[-0.26 -0.09]	ns	[-0.08 0.06]	4.00E-03	[-0.18 -0.03]	4.06E-08	[-0.37 -0.18]
'FAM26F'	3.67E-02	[0.02 0.65]	1.80E-03	[0.11 0.48]	1.50E-06	[0.23 0.55]	2.15E-14	[0.77 1.28]
'FPR1'	9.89E-04	[0.26 1.02]	7.98E-05	[0.19 0.56]	1.06E-09	[0.29 0.56]	6.62E-09	[0.47 0.93]
'FPR2'	ns	[-0.02 0.66]	1.04E-04	[0.11 0.33]	1.21E-02	[0.03 0.25]	4.47E-07	[0.46 1.02]
'GBP6'	9.62E-04	[0.10 0.40]	7.03E-08	[0.16 0.33]	1.73E-04	[0.13 0.41]	9.74E-05	[0.41 1.23]
'GNGT2'	1.17E-03	[0.12 0.48]	6.21E-05	[0.12 0.34]	2.74E-10	[0.19 0.36]	7.25E-08	[0.31 0.66]
'GPR18'	ns	[-0.05 0.32]	5.81E-03	[0.05 0.29]	1.89E-12	[0.34 0.60]	1.06E-10	[0.53 0.99]
'HERC6'	1.78E-02	[0.04 0.46]	ns	[-0.15 0.10]	ns	[-0.03 0.32]	4.17E-05	[0.19 0.53]
'HP'	6.47E-03	[0.12 0.73]	ns	[-0.08 0.45]	9.24E-07	[0.33 0.77]	ns	[-0.32 0.82]
'IFI44'	1.48E-03	[0.19 0.79]	2.44E-02	[0.03 0.42]	1.83E-03	[0.10 0.42]	3.32E-08	[0.43 0.89]
'IFIT2'	2.06E-04	[0.23 0.74]	5.60E-05	[0.20 0.56]	3.97E-02	[0.01 0.32]	1.30E-11	[0.45 0.80]
'IRAK3'	2.44E-02	[0.04 0.62]	2.82E-26	[0.69 0.98]	4.33E-14	[0.46 0.78]	1.15E-08	[0.43 0.87]
'ISG15'	5.01E-03	[0.13 0.71]	1.53E-02	[-0.63 -0.07]	3.32E-07	[-0.77 -0.34]	ns	[-0.48 0.01]
'ITGAL'	2.57E-04	[0.30 1.00]	3.89E-05	[0.23 0.64]	3.50E-05	[0.17 0.48]	1.13E-18	[0.72 1.11]
'MARCO'	3.63E-08	[0.98 2.02]	5.40E-03	[0.09 0.49]	ns	[-0.06 0.46]	3.16E-04	[0.31 1.05]
'MX1'	1.28E-03	[0.22 0.89]	1.05E-02	[0.07 0.50]	ns	[-0.31 0.09]	1.27E-03	[0.15 0.60]
'NFKBIZ'	1.72E-03	[-0.63 -0.15]	ns	[-0.12 0.34]	2.05E-04	[0.17 0.56]	1.87E-05	[0.23 0.61]
'PSTPIP2'	ns	[-0.16 0.37]	5.56E-04	[0.09 0.33]	ns	[-0.09 0.14]	2.02E-02	[0.03 0.37]
'PYHIN1'	6.80E-03	[0.10 0.59]	2.79E-05	[0.19 0.52]	3.45E-18	[0.58 0.92]	1.89E-13	[0.71 1.20]
'RSAD2'	3.55E-03	[0.17 0.86]	ns	[0.00 0.39]	1.07E-03	[0.12 0.48]	3.85E-07	[0.35 0.79]
'STAT1'	1.64E-03	[0.12 0.52]	ns	[-0.11 0.18]	2.18E-03	[0.07 0.33]	1.20E-10	[0.36 0.66]
'STAT2'	5.44E-06	[0.21 0.51]	ns	[-0.05 0.08]	8.32E-07	[0.10 0.22]	3.03E-06	[0.13 0.31]
'TLR2'	1.10E-04	[0.25 0.76]	5.06E-09	[0.32 0.63]	5.33E-08	[0.21 0.45]	2.57E-10	[0.48 0.91]
'TUBA4A'	3.91E-02	[-0.27 -0.01]	1.88E-04	[0.16 0.51]	ns	[-0.19 0.12]	2.90E-03	[-0.42 -0.09]
'XAF1'	1.35E-03	[0.18 0.73]	1.99E-05	[0.24 0.65]	1.34E-03	[0.10 0.39]	1.80E-10	[0.43 0.81]

Table S1: M_1-associated gene set enrichment: Reported are all genes (out of 36 total) having statistically significant differential expression (samples segregated based on median EMT score) across TCGA colorectal adenocarcinoma (Adeno COL; n=286),

prostate adenocarcinoma (Adeno PCA; n=497), invasive breast cancer (BCA invasive; n=1097), and lung adenocarcinoma (Lung Adeno; n=515). For each gene, an unpaired t-test at significance level α=0.05 was performed under the null hypothesis that EMT-high and EMT-low mean gene expression signatures are equal. Blue p-values correspond to the M_1-associated genes that are enriched in EMT-score-high tumors, whereas red p-values correspond to the M_1-associated genes that are enriched in EMT-score-low tumors. ‘ns’ stands for non-significant.
